# Burden of obesity in gastrointestinal and liver diseases

**DOI:** 10.1002/ueg2.12302

**Published:** 2022-08-31

**Authors:** Zeljko Krznaric

**Affiliations:** ^1^ Department of Gastroenterology, Hepatology and Nutrition Clinical Hospital Centre & School of Medicine Zagreb Zagreb Croatia

The obesity epidemic places a heavy burden on individuals, society and GI health care providers in particular. Nearly 1 in 4 European individuals are considered obese and will develop associated disorders such as diabetes, cardiovascular and liver disease and cancer that will reduce life expectancy. There is a need to provide the GI community with guidance how to care for these individuals. That is why UEG and ESPEN embarked on a collaborative project in the topic of nutrition in gastroenterology, and found a common interest in providing a guideline on obesity in gastrointestinal and liver diseases.

ESPEN brought together numerous experts responsible for the evaluation of aspects related to the epidemiology and aetiology of overweight and obesity, as well as pathophysiology, clinical manifestations, diagnostic procedures with a special interest in nutritional interventions as well as all available therapeutic.^1^

The guideline ‘European Guideline on Obesity care in patients with gastrointestinal and liver diseases—joint ESPEN/UEG guideline’, included in this issue of the UEG Journal, is the result of this enthusiastic collaboration.^1^ It is well known that obesity is a complex multifactorial disease characterised by the excessive accumulation of adipose tissue and low‐grade inflammation which presents a health risk leading and leads to the development of associated chronic diseases of the digestive system. For example, obesity related fatty liver disease causes progressive changes in the liver parenchyma and puts patients on a higher risk for hepatocellular cellular carcinoma, in addition to the risk for malignancies elsewhere in the digestive system.

In some diseases, such as for inflammatory bowel disease (IBD), the situation has changed dramatically over the past few decades, with a significant increase in the number of IBD patients who are overweight and suffer from obesity‐related problems.^2^, ^3^ Crohn's disease, which was previously almost synonymous with malnutrition, is now characterised by overweight and obesity as well as sarcopenic obesity as a sign of malnutrition and loss of muscle mass.^1^, ^4^ Along with all of its well‐known consequences, obesity in IBD patients significantly compromises the application of most of new treatment modalities, such as biological therapy.^5^, ^6^, ^7^ Obesity can also compromise the results of surgical treatment such as bariatric surgery in IBD.^8^, ^9^

In some cases, it is necessary to strive for weight loss first before to go for biological therapy or IBD surgery. The problem is slightly less pronounced in ulcerative colitis.^1^

Overweight and obesity are becoming an increasingly common problem even in patients with celiac disease,^10^ as well as in patients with irritable bowel syndrome, which raises questions whether new therapeutic approaches such as microbiota manipulation may be a panacea for these issues.^1^

The connection between gastroesophageal reflux disease and obesity is well known, and the interventions related to this disease range from lifestyle changes and weight loss diets to bariatric surgery.^1^

Non‐alcoholic fatty liver disease (NAFLD) and metabolic‐associated fatty liver disease (MAFLD) are undoubtedly the most common GI pathologies associated with overweight and obesity. In an effort of defining an umbrella term, MAFLD seems like a reasonable proposal.^11^

Numerous diagnostic approaches for assessment changes of liver parenchyma and liver function have been developed. These range from application of biomarkers to advanced ultrasound methods for assessing the degree of fatty infiltration of the liver, or more advanced stages like fibrosis^12^

From a therapeutic perspective this guideline emphasises important role of all known therapeutic options from dietetic interventions and lifestyle changes with an accent on physical activity to the bariatric surgery, and recently, medical therapy (anti‐obesity drugs) in the obese patients with NAFLD/MAFLD.^1^, ^13^, ^14^ It has been shown that obesity curtailing interventions improve histologic features of NAFLD.^14^

A recent prospective cohort study confirmed that morbid obesity is associated with mortality outside but also on the liver transplantation waiting lists.^15^

In conclusion, we can say that raising awareness about on overweight and obesity in gastroenterology is of particular interest.

The first step that should be taken is the assessment of the nutritional status in all clinical entities. After that, special attention should be paid to the problem of obesity, which often goes unnoticed in routine clinical practice, and which can significantly affect the clinical course of the disease and treatment outcomes of various GI diseases.

Nowadays, we have a plethora of treatment options from lifestyle changes, nutritional interventions and diets, drug therapy to endoscopic and surgical approaches. This guideline provides therapeutic perspective but also recognizes the fact that there are still many unanswered questions.^1^

It may be a bit controversial, but gastroenterologists will also have to move in the direction of primary prevention, and it is particularly important to consider interventions that target obesity prevention in GI patients.

A holistic approach is needed for the prevention of obesity, and we will have to devote our knowledge and skills to educate the wider population. Without the joint action of the community and the entire medical profession, it will not be possible to prevent or even solve obesity related challenges.

## AUTHOR CONTRIBUTION

The author contributed to the conceptualisation, writing and revision of the manuscript. The author approved the final version of the manuscript.

## CONFLICT OF INTEREST

The author has no conflict of interest to declare regarding this manuscript.

## Data Availability

Data sharing is not applicable to this article as no new data were created or analysed in this study.
